# P-08 - ASSOCIATIONS BETWEEN ANTIDEPRESSANT PRESCRIBING AND ANTIBIOTIC RESISTANCE IN ESCHERICHIA COLI URINARY TRACT INFECTIONS IN SCOTLAND

**DOI:** 10.1093/pubmed/fdag046.079

**Published:** 2026-07-09

**Authors:** Dr Laura Ciaccio, Dr Benjamin Parcell, Dr Huan Wang, Dr Atanu Bhattacharjee, Janathan Danial, Frances Kerr, Jo McEwen, Aidan Morrison, Deirdre O'Driscoll, Andrew Seaton, Dr Charis Marwick, Dr Karen Barnett

**Affiliations:** Division of Population Health and Genomics, University of Dundee School of Medicine; Division of Population Health and Genomics, University of Dundee School of Medicine; Division of Population Health and Genomics, University of Dundee School of Medicine; Division of Population Health and Genomics, University of Dundee School of Medicine; ARHAI Scotland, NHS National Services Scotland; NHS Healthcare Improvement Scotland; NHS Tayside; Public Health Scotland; NHS Greater Glasgow and Clyde; NHS Greater Glasgow and Clyde; Division of Population Health and Genomics, University of Dundee School of Medicine; Division of Population Health and Genomics, University of Dundee School of Medicine

## Abstract

**Background:**

Preclinical evidence demonstrates that antidepressant drugs possess antibiotic properties that can induce antibiotic resistance, but no population-based studies have examined effects in clinical infections. This could have important implications for prescribing guidelines and patient care.

**Objectives:**

This study examined associations between antidepressants and antibiotic resistance in Escherichia coli (E. coli) urinary tract infections (UTIs) in Scotland.

**Methods:**

*E. coli* urinary isolates in Tayside (population=414,000), Scotland from individuals ≥16 years old from January 2019 to December 2023 were included. *E. coli* UTI episodes were linked to demography, healthcare, and community prescribing data. Univariate and multivariable logistic regression assessed associations between antidepressant use in the year prior to UTI and the odds of resistant and multidrug resistant (MDR) *E. coli* isolates. Covariates included age; sex; deprivation (Scottish Index of Multiple Deprivation quintile); comorbidity (Elixhauser unweighted score); infection source (community or hospital); year of episode; catheter use; number of hospital admissions; previous *E. coli* UTI; previous urinary procedure; and prescriptions for proton pump inhibitors (PPIs), beta-blockers, statins, and relevant antibiotics.

**Results:**

There were 60,187 *E. coli* UTI episodes (in 30,887 individuals) during the study period. Antidepressants were recorded in 37.6% of episodes (n=22,061). MDR rates were higher in episodes in people with antidepressant prescriptions compared to those without (39.0% vs. 34.5%). In multivariable analysis, antidepressants increased the odds of any resistance (Adjusted OR 1.07 [95%CI1.03-1.11]) and MDR (Adjusted OR 1.10 [95%CI 1.06-1.15]). Other factors associated with increased odds of MDR in multivariable analysis included certain age groups; male sex; increased comorbidity; hospital-acquired infections; catheter use; prior hospital admissions; prior UTI; PPIs; and prior antibiotic prescriptions.

**Conclusions:**

Antidepressants are associated with statistically significant increased odds of antibiotic resistance in *E.coli* UTI episodes. Targeted antibiotic prescribing, urine sample submission, and minimising unnecessary antibiotic exposure may be important for people prescribed antidepressants.

